# Multiscale Traffic Dynamics Representation for Forecasting via MEMD-Guided Dual-Branch Recurrent Networks

**DOI:** 10.3390/s26113369

**Published:** 2026-05-26

**Authors:** Yichen Qian, Taiming Kang, Shengduo Zhang, Chaoneng Li, Xiaolong Wang, Shuxu Zhao

**Affiliations:** School of Electronic and Information Engineering, Lanzhou Jiaotong University, Lanzhou 730070, China; 20233202117@stu.lzjtu.edu.cn (Y.Q.); 20233202111@stu.lzjtu.edu.cn (T.K.); 20233202133@stu.lzjtu.edu.cn (S.Z.); wxlong25300@163.com (X.W.); zhaosx_2012@163.com (S.Z.)

**Keywords:** traffic flow forecasting, multivariate empirical mode decomposition (MEMD), multiscale temporal modeling, dual-branch recurrent network

## Abstract

Traffic flow forecasting remains challenging because raw traffic flow observations often contain mixed temporal patterns, including slowly varying trends and fast local fluctuations. To address this issue, this paper proposes a Multivariate Empirical Mode Decomposition (MEMD)-guided dual-branch recurrent framework for multistep point forecasting. Specifically, MEMD is used as an alignment-preserving multivariate decomposition mechanism to obtain frequency-aligned components, which are then reconstructed into low-frequency trend and high-frequency residual components. The trend component is modeled by a Long Short-Term Memory (LSTM) branch to capture smooth long-term evolution, while the residual component is learned by a Bidirectional Gated Recurrent Unit (Bi-GRU) branch to characterize short-term oscillatory dynamics. A lightweight fusion head is then used to integrate the two branch-specific representations for final prediction. Experiments on PeMS04 and PeMS08, two traffic datasets derived from the California Department of Transportation Performance Measurement System, show that the proposed method achieves competitive performance across mean absolute error (MAE), root mean square error (RMSE), and mean absolute percentage error (MAPE), reaching 19.67/31.59/12.95% on PeMS04 and 15.51/24.43/9.86% on PeMS08. Compared with representative recent baselines, the proposed method achieves competitive results, with relative gains reaching 5.89% on PeMS04 and 5.35% on PeMS08 in selected metric-wise comparisons. These results indicate that MEMD-guided trend–residual representation learning can improve multistep traffic flow forecasting.

## 1. Introduction

Traffic flow forecasting is a fundamental task in intelligent transportation systems because it supports signal control, route guidance, congestion mitigation, and large-scale traffic management. In this study, traffic flow measurements refer to time-series observations collected by road sensors, such as flow and speed. Despite substantial progress, accurate multistep prediction remains challenging [1], because traffic observations often mix slowly varying background demand, recurrent mobility patterns, and short-lived local disturbances, making the resulting sequences nonlinear and nonstationary [2,3]. A diagnostic analysis on PeMS04 further shows that mixed-scale dynamics substantially affect forecasting errors. The raw-input baseline yields mean absolute error/root mean square error (MAE/RMSE) values of 15.39/26.28 in stable intervals, but the errors increase to 41.24/56.34 during rapid transitions and reach 41.96/57.77 in peak intervals. With trend–residual reconstruction, the proposed framework reduces MAE, RMSE, and mean absolute percentage error (MAPE) by 22.91%, 20.01%, and 25.44% during rapid-transition intervals, indicating that organizing multiscale components before prediction improves forecasting stability.

Existing traffic forecasting methods include statistical models, recurrent networks, graph-based models, and attention-based architectures. Statistical models are efficient but often rely on restrictive assumptions [3]. Recurrent architectures such as Long Short-Term Memory (LSTM) and Gated Recurrent Unit (GRU) improve temporal modeling from historical observations [4], while graph-based and attention-based models strengthen spatial interaction modeling and long-range dependency learning [5,6]. However, many methods still model raw traffic sequences directly and require a single predictor to absorb multiple temporal regimes into one latent representation. As a result, slow structural evolution and fast local fluctuations are often learned together, although they reflect markedly different temporal behaviors. This mixed-scale representation may weaken multistep forecasting stability [7].

For traffic flow data, the challenge is not only architectural but also representational. When components with distinct temporal scales are entangled at the input level, local representation errors may accumulate over longer horizons [8]. This suggests that improving forecasting performance requires not only a stronger predictor, but also a more suitable organization of traffic flow observations before prediction.

Motivated by this observation, we propose a decomposition-guided forecasting framework that reorganizes multiscale traffic observations before recurrent learning. The framework uses Multivariate Empirical Mode Decomposition (MEMD) to obtain aligned multiscale components across variables [9]. Rather than serving as a standalone preprocessing tool or a new decomposition algorithm, MEMD is used as a multivariate alignment mechanism for constructing forecasting-oriented trend–residual representations. The reconstructed trend and residual representations are modeled by component-specific recurrent branches, with LSTM capturing trend continuity and Bidirectional Gated Recurrent Unit (Bi-GRU) capturing rapidly changing residual patterns. The branch-specific representations are then integrated through a lightweight fusion head for multistep forecasting [10].

By reconstructing persistent evolution and transient variation before prediction, the proposed framework avoids forcing heterogeneous temporal behaviors into a single latent state. Experiments on PeMS04 and PeMS08, two benchmark datasets derived from the California Department of Transportation Performance Measurement System (Caltrans PeMS), show that the proposed framework achieves competitive forecasting performance against representative baselines. These results suggest that trend–residual representation learning provides a useful way to organize heterogeneous temporal patterns before multistep prediction.

The contributions of this study are summarized as follows.

- A MEMD-guided trend–residual framework is used to reconstruct frequency-aligned components into compact trend and residual representations.
- A heterogeneous dual-branch architecture models the reconstructed components with component-specific encoders: LSTM for trend evolution and Bi-GRU for residual fluctuations.
- Experiments and ablations on PeMS04 and PeMS08 show competitive results, suggesting that the gains mainly come from trend–residual representation learning and component-specific temporal modeling.

## 2. Related Work

Traffic flow forecasting has evolved from classical statistical models to deep spatio-temporal representation learning. Early methods, such as AutoRegressive Integrated Moving Average (ARIMA) and Kalman filtering, are efficient and interpretable but rely on approximate stationarity or near-linear dynamics, limiting their robustness under nonlinear traffic patterns and external disturbances [11,12]. Recurrent models, including LSTM and GRU, improve temporal dependency modeling from historical observations [13], while graph-based methods further capture sensor–network interactions, including Spatio-Temporal Graph Convolutional Network (STGCN) [14], Diffusion Convolutional Recurrent Neural Network (DCRNN) [15], Graph WaveNet [16], Temporal Graph Convolutional Network (T-GCN) [17], Spatial–Temporal Synchronous Graph Convolutional Network (STSGCN) [18], and Adaptive Graph Convolutional Recurrent Network (AGCRN) [19]. Attention- and Transformer-based models have also been developed for long-range dependency modeling, including Attention-based Spatial–Temporal Graph Convolutional Network (ASTGCN) [20], Graph Multi-Attention Network (GMAN) [21], Traffic Transformer [22], Autoformer [23], Propagation Delay-Aware Dynamic Long-Range Transformer (PDFormer) [24], and Spatio-Temporal Adaptive Embedding Transformer (STAEformer) [25], with related progress summarized in meta-learning-oriented work and surveys [26,27,28]. Recent work has also emphasized the need to understand why traffic forecasting models make certain predictions, rather than evaluating them only by aggregate accuracy. Studies using extreme gradient boosting with Shapley additive explanations (XGBoost–SHAP) have identified influential road links and show that predictive influence is not always limited to adjacent links [29]. Explainable artificial intelligence (XAI)-based traffic-flow analysis has improved the transparency of forecasting results for traffic management applications [30], while explainable analysis has also been used to reveal sensitive spatial units and failure modes in mobile traffic forecasting [31].

Despite these advances, many forecasting frameworks still treat multivariate traffic observations as a single entangled sequence, leaving the underlying multiscale structure to be learned implicitly [32]. Since traffic measurements contain both short-term fluctuations and longer-term trends, such entanglement can introduce scale ambiguity and make stable cross-variable representation learning more difficult in multivariate settings [33]. Decomposition-based forecasting addresses this issue by separating nonstationary signals into simpler components before prediction. Existing studies have applied Variational Mode Decomposition (VMD) and Empirical Mode Decomposition (EMD) to traffic forecasting [34], with related extensions using singular spectrum analysis [35], adversarial learning for uncertainty modeling [36], and gray models for interpretable forecasting [37].

However, when decomposition is performed independently for each variable, the resulting modes may be inconsistent across channels, weakening the representation of synchronous cross-variable variations. MEMD provides a multivariate alternative by jointly decomposing coupled signals so that extracted modes remain aligned across variables through hyperspherical projections [38]. Its filter-bank property further supports consistent multiscale modeling from aligned time–frequency representations [39]. In this study, MEMD is not introduced as a new decomposition algorithm; instead, it serves as an alignment mechanism for forecasting-oriented trend–residual reconstruction. Rather than modeling raw sequences directly or concatenating decomposed modes without structure, the proposed framework reconstructs aligned components into low-frequency trend and high-frequency residual representations and assigns them to branch-specific recurrent encoders. This design organizes heterogeneous temporal patterns before prediction and provides a more stable representation basis for multistep traffic flow forecasting.

## 3. Methodology

### 3.1. Overview of the Proposed Framework

**Figure 1:**
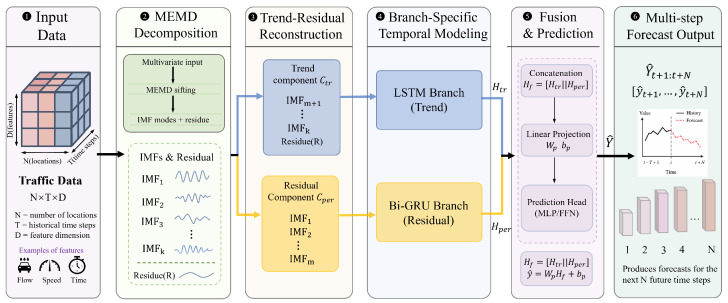
Architectureof the MEMD-LSTM-Bi-GRU framework.

To model heterogeneous temporal variations in multivariate traffic data, we construct a heterogeneous deep learning framework based on MEMD-guided trend–residual representation. As illustrated in Figure 1, the proposed architecture processes the multivariate input tensor $X\in R^{N\times T\times D}$ through a decomposition-guided dual-branch pipeline, where the main stages include MEMD decomposition, trend–residual reconstruction, branch-specific temporal modeling, and fusion-based prediction.

Figure 1 illustrates the workflow of the proposed MEMD-guided dual-branch recurrent forecasting framework. Given a multivariate traffic input tensor $X\in R^{N\times T\times D}$, where *N*, *T*, and *D* denote the number of locations, the historical observation length, and the feature dimension, respectively, MEMD first decomposes the input window into Intrinsic Mode Function (IMF) modes and a residual component. These decomposed modes are then reconstructed into two signals with distinct temporal roles: a low-frequency trend component $C_{tr}$, which preserves the slowly varying baseline evolution and structural continuity of traffic states, and a high-frequency residual component $C_{per}$, which captures short-term oscillations, local disturbances, and rapid temporal variations. The reconstructed trend component is subsequently fed into an LSTM branch to model long-range temporal dependencies and stable state evolution, whereas the residual component is processed by a Bi-GRU branch to encode transient fluctuations and local temporal changes from both temporal directions within the observation window. The resulting branch-specific representations $H_{tr}$ and $H_{per}$ are then integrated through a lightweight fusion head based on concatenation and projection to produce the final multistep forecasts ${\hat{Y}}_{t+1:t+N}$. In this way, the framework organizes trend-dominated and fluctuation-dominated information into separate modeling paths, which helps the network learn multiscale traffic patterns in a more targeted manner for multistep forecasting. It should be noted that the current framework focuses on multiscale temporal representation learning and does not include an explicit graph convolution, road-network adjacency matrix, distance matrix, or sensor-to-sensor message-passing module. Spatial dependency modeling is therefore not the main contribution of this study.

### 3.2. Spectral Decomposition via MEMD

Standard univariate decomposition often fails to preserve the cross-channel correlations in multimodal data, leading to mode misalignment where temporally related variations are scattered across disparate frequency scales. To address this, we utilize Multivariate Empirical Mode Decomposition (MEMD), which generalizes the sifting process to multivariate signals and helps preserve aligned time–frequency representations across channels. In this study, MEMD is applied once to each historical multichannel input window, rather than independently to individual sensors or variables. For each supervised sample $\left(X_{i},Y_{i}\right)$, only the input window $X_{i}=\left[x_{i-T+1},\ldots ,x_{i}\right]$ is decomposed, while the future target window $Y_{i}=\left[x_{i+1},\ldots ,x_{i+H}\right]$ and observations outside the current input window are excluded to avoid information leakage. Specifically, the traffic-flow measurements of all sensors within the window are arranged as $V_{i}\in R^{T\times N}$, where *T* is the historical observation length and *N* is the number of sensors. Thus, the MEMD input contains 307 channels for PeMS04 and 170 channels for PeMS08. Under this setting, the extracted IMFs are generated under the same projection directions and sifting process, which naturally preserves mode alignment across variables.

Unlike EMD, which identifies local extrema, MEMD projects the multivariate signal $v\left(t\right)$ onto a set of direction vectors d sampled from an $\left(n-1\right)$-dimensional hypersphere. To obtain a more uniform coverage of the projection space, we use the Hammersley sequence to generate the direction vectors.

For each direction vector $d_{k}$ (where k=1,…,K), the projection of the multivariate signal is calculated as $p_{\theta_{k}}\left(t\right)=v\left(t\right)\cdot d_{k}$. The multivariate local mean m(t) is then estimated by averaging the envelopes of these projections:

(1) $$m\left(t\right)=\frac{1}{K}\sum_{k=1}^{K}e_{\theta_{k}}\left(t\right),$$

where $e_{\theta_{k}}\left(t\right)$ denotes the envelope of the projected signal $p_{\theta_{k}}\left(t\right)$, derived via cubic spline interpolation of its extrema. By iteratively subtracting this local mean ($v_{new}\left(t\right)=v\left(t\right)-m\left(t\right)$) until the stopping criterion is met, MEMD decomposes the complex signal into a set of Intrinsic Mode Functions (IMFs), $c_{i}\left(t\right)$, and a final monotonic residue r(t):

(2) $$v\left(t\right)=\sum_{i=1}^{M}c_{i}\left(t\right)+r\left(t\right).$$

The number of IMFs *M* is adaptively determined by the MEMD sifting process rather than manually fixed. All extracted IMFs are retained and grouped into trend or residual components according to their zero-crossing characteristics, so reconstruction does not rely on a predefined number of modes. Because the same set of direction vectors is applied to all channels simultaneously, the resulting IMFs are aligned in frequency across all variables. Rather than physically decoupling traffic dynamics, this alignment helps separate high-frequency fluctuations from low-frequency structural components while preserving cross-variable temporal consistency.

### 3.3. Component Reconstruction and Heterogeneous Modeling

Directly modeling raw IMFs is computationally inefficient and may lead to overfitting due to spectral redundancy. Therefore, we introduce a reconstruction strategy based on Zero-Crossing Rate (ZCR), followed by component-specific neural modeling.

#### 3.3.1. Adaptive Component Reconstruction of MEMD Modes

We use the Zero-Crossing Rate (ZCR) to characterize the oscillatory intensity of each IMF component. For an IMF component $c_{i}$, the ZCR is defined as the normalized count of sign changes:

(3) $$ZCR_{i}=\frac{1}{T-1}\sum_{t=1}^{T-1}1\left(c_{i}\left(t\right)c_{i}\left(t+1\right)<0\right),$$

where 1{·} is the indicator function. This metric effectively quantifies the oscillatory intensity of each mode. Rather than employing a heuristic cutoff, we determine an adaptive threshold τ via K-means clustering of the ZCR distribution. This divides the intrinsic modes into two spectral clusters:

Residual Component ($C_{per}$): Defined as $\sum_{i\in \Omega_{high}}c_{i}\left(t\right)$, where $\Omega_{high}=\left\{i|ZCR_{i}>\tau \right\}$. This component contains high-ZCR IMFs and represents rapid local fluctuations and short-term deviations from the traffic baseline.

Trend Component ($C_{tr}$): Defined as $\sum_{i\in \Omega_{low}}c_{i}\left(t\right)+r\left(t\right)$, where $\Omega_{low}=\left\{i|ZCR_{i}\leq \tau \right\}$. This component contains low-ZCR IMFs and the residue, representing slowly varying baseline evolution in the traffic sequence.

The two-component reconstruction retains all extracted IMFs, with the high-frequency and low-frequency IMF index sets satisfying:

(4) $$\Omega_{high}\cup \Omega_{low}=\left\{1,2,\ldots ,M\right\},\;\Omega_{high}\cap \Omega_{low}=\emptyset .$$

Therefore, the reconstructed components satisfy:

(5) $$C_{tr}\left(t\right)+C_{per}\left(t\right)=\sum_{i\in \Omega_{low}}c_{i}\left(t\right)+r\left(t\right)+\sum_{i\in \Omega_{high}}c_{i}\left(t\right)=\sum_{i=1}^{M}c_{i}\left(t\right)+r\left(t\right)=v\left(t\right).$$

Equations (4) and (5) show that the two reconstructed components retain all extracted IMFs and the residue, thereby preserving the decomposed signal at the reconstruction level. The effectiveness of this compact grouping is further evaluated through the grouping-strategy ablation in Section 5. The reconstructed components are not direct labels of specific traffic causes, such as incidents, weather effects, or predefined daily patterns. Instead, they provide flow-derived descriptions of temporal behavior: the trend component reflects slowly varying baselines, recurrent demand levels, and sustained congestion states, whereas the residual component captures rapid departures from the baseline that may correspond to abrupt changes, short-term oscillations, and local fluctuations in traffic flow.

The reconstruction procedure is summarized in Algorithm 1.
**Algorithm 1** MEMD-Based Component Reconstruction**Input:** 
Historical multichannel sensor-flow input window $V_{i}=X_{i}\in R^{T\times N}$, where N=307 for PeMS04 and N=170 for PeMS08; number of projection directions $N_{v}$.**Output:** 
Reconstructed residual component $C_{\mathrm{per}}$; reconstructed trend component $C_{\mathrm{tr}}$.  1:Generate direction set $V=\{v^{\left(1\right)},\ldots ,v^{\left(N_{v}\right)}\}$ on $S^{N-1}$ via the Hammersley sequence  2:$\left\{U_{1},\ldots ,U_{M},R\right\}\leftarrow \mathrm{MEMD}\left(X_{i};V\right)$  3:**for** k=1 to *M* **do**  4:   $s_{k}\leftarrow \mathrm{ZCR}\left(U_{k}\right)$  5:**end for**  6:Partition ${\left\{U_{k}\right\}}_{k=1}^{M}$ into two groups by K-means on ${\left\{s_{k}\right\}}_{k=1}^{M}$  7:Identify $I_{\mathrm{per}}$ as the cluster with the larger ZCR centroid  8:Set $I_{\mathrm{tr}}\leftarrow \left\{1,\ldots ,M\right\}\setminus I_{\mathrm{per}}$  9:$C_{\mathrm{per}}\leftarrow \sum_{k\in I_{\mathrm{per}}}U_{k}$10:$C_{\mathrm{tr}}\leftarrow \sum_{k\in I_{\mathrm{tr}}}U_{k}+R$11:**return** $C_{\mathrm{per}},C_{\mathrm{tr}}$

**Figure 2:**
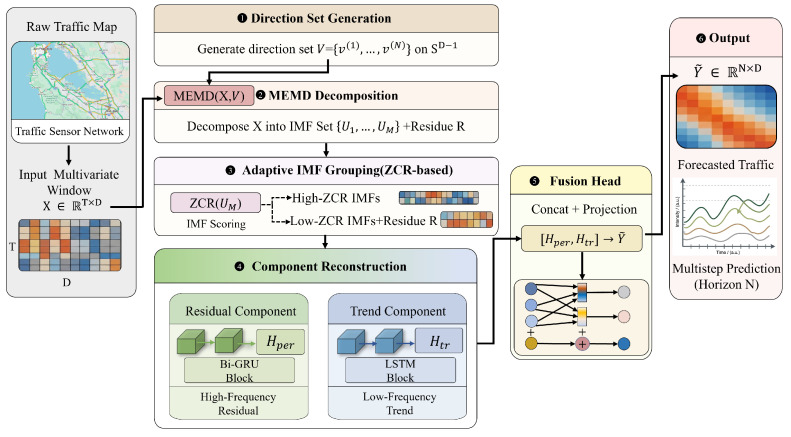
MEMD-based component reconstruction and branch assignment.

Upon applying Algorithm 1, the multivariate traffic window is reorganized into two frequency-related components. High-ZCR IMFs are assigned to the residual component, while low-ZCR IMFs together with the residue are assigned to the trend component. As illustrated in Figure 2, this procedure connects MEMD decomposition, ZCR-based IMF grouping, component reconstruction, and branch-specific modeling within a unified forecasting workflow. The trend component preserves slowly varying baseline evolution, whereas the residual component captures short-term fluctuations and local deviations. These two components provide structured inputs for the subsequent LSTM and Bi-GRU branches.

#### 3.3.2. Computational Cost Discussion

MEMD adds preprocessing cost due to multivariate projection, envelope interpolation, and iterative sifting. This cost is more noticeable because MEMD is applied to multichannel sensor-flow windows rather than to a single univariate sensor sequence. To reduce this overhead, we perform MEMD-based reconstruction offline and cache the reconstructed trend and residual components before neural network training. Here, offline reconstruction means that MEMD is precomputed separately for each historical input window and cached before training; it does not mean decomposing the entire time series before the train/validation/test split. During training, the LSTM–Bi-GRU predictor only uses the cached components $C_{\mathrm{tr}}$ and $C_{\mathrm{per}}$, so MEMD is not repeated in each epoch. Nevertheless, the efficiency of MEMD-based reconstruction remains a limitation for large-scale online deployment. Future work will explore incremental MEMD, parallelized sifting, and periodic cache updates to support efficient reconstruction under streaming traffic conditions.

#### 3.3.3. Trend Representation Learning Based on LSTM

The trend component characterizes slowly varying traffic evolution, so an LSTM encoder is adopted to model the reconstructed trend sequence $C_{tr}$ [40]. For the trend input $x_{t}^{tr}$, the LSTM updates are given by

(6) $$f_{t}=\sigma \left(W_{f}\cdot \left[h_{t-1},x_{t}^{tr}\right]+b_{f}\right),$$

(7) $$i_{t}=\sigma \left(W_{i}\cdot \left[h_{t-1},x_{t}^{tr}\right]+b_{i}\right),$$

(8) $${\tilde{C}}_{t}=\tanh\left(W_{c}\cdot \left[h_{t-1},x_{t}^{tr}\right]+b_{c}\right),$$

(9) $$C_{t}=f_{t}⊙C_{t-1}+i_{t}⊙{\tilde{C}}_{t},$$

(10) $$o_{t}=\sigma \left(W_{o}\cdot \left[h_{t-1},x_{t}^{tr}\right]+b_{o}\right),$$

(11) $$h_{t}=o_{t}⊙\tanh\left(C_{t}\right).$$

Here, σ denotes the sigmoid activation function, ⊙ denotes the Hadamard product, and ${\tilde{C}}_{t}$ is the candidate cell state. The gated cell update enables the LSTM branch to retain low-frequency temporal continuity in $C_{tr}$.

#### 3.3.4. Residual Dynamics Modeling Based on Bi-GRU

The residual component $C_{\mathrm{per}}$ contains rapid transitions, local oscillations, and short-term deviations from the traffic baseline. We therefore use a Bidirectional Gated Recurrent Unit (Bi-GRU) encoder to model residual dynamics within the observed input window [41]. The bidirectional structure allows the residual representation at each position to incorporate contextual information from both earlier and later observations within the same historical window, without accessing future prediction targets.

The forward hidden state ${\vec{h}}_{t}$ encodes the residual sequence in chronological order, while the backward hidden state ${\overset{\leftarrow }{h}}_{t}$ encodes the same historical input window in reverse order. Here, the reverse direction does not access future prediction targets; it only uses later observations that are already contained within the historical input window available at the forecasting time. This window-level bidirectional encoding is therefore used to obtain a more complete representation of short-term residual fluctuations within the observed history, rather than to introduce noncausal information from the prediction horizon. For the reconstructed residual component $C_{\mathrm{per}}$, the Bi-GRU branch processes the component-specific input $x_{t}^{\mathrm{per}}$ rather than the raw traffic input or the trend component. The bidirectional updates are formulated as follows:

(12) $${\vec{h}}_{t}=GRU\left(x_{t}^{per},{\vec{h}}_{t-1}\right),$$

(13) $${\overset{\leftarrow }{h}}_{t}=GRU\left(x_{t}^{per},{\overset{\leftarrow }{h}}_{t+1}\right).$$

Therefore, the two branches differ not only in their recurrent structures, but also in their component-specific inputs: the LSTM branch models $C_{tr}$, whereas the Bi-GRU branch models $C_{per}$.

The resulting representation of the residual component, $H_{per}$, is derived via the concatenation of these directional vectors:

(14) $$H_{per}=\left[{\vec{h}}_{t}⊕{\overset{\leftarrow }{h}}_{t}\right].$$

This bidirectional fusion allows each residual representation to use contextual information from both earlier and later positions within the observed input window, thereby improving the modeling of short-term oscillations and local residual peaks without using future target values.

### 3.4. Adaptive Fusion and Optimization

The independent feature representations extracted by the LSTM ($H_{tr}$) and Bi-GRU ($H_{per}$) branches characterize complementary temporal aspects of the reconstructed traffic flow observation. To generate a unified prediction, these component-specific representations are integrated through a nonlinear fusion mechanism.

#### 3.4.1. Lightweight Fusion and Prediction Head

The trend and residual representations $H_{tr}$ and $H_{per}$ are fused by a lightweight prediction head. The joint representation is first formed by concatenation and then mapped to the forecast space:

(15) $$Z=\mathrm{ReLU}\left(W_{c}\left[H_{\mathrm{tr}}⊕H_{\mathrm{per}}\right]+b_{c}\right),$$

(16) $$\hat{Y}=W_{o}Z+b_{o}.$$

Here, ⊕ denotes concatenation, and ReLU denotes the rectified linear unit activation function. The lightweight head preserves component-specific information before prediction and keeps the output module from dominating the decomposition and branch-modeling effects. The ReLU activation introduces a nonlinear transformation in the fusion head, enabling flexible integration of the trend and residual representations before they are mapped to the prediction horizon.

#### 3.4.2. End-to-End Optimization

To train this heterogeneous architecture, we optimize the model with a Mean Squared Error (MSE)-based objective and an $L_{2}$ regularization term:

(17) $$J\left(\Theta \right)=\frac{1}{N}\sum_{i=1}^{N}{\left(y_{i}-{\hat{y}}_{i}\right)}^{2}+\lambda {∥\Theta ∥}_{2}^{2}.$$

Here, the first term measures the discrepancy between the ground-truth traffic flow $y_{i}$ and the prediction ${\hat{y}}_{i}$, while the second term applies an $L_{2}$ penalty to the model parameters Θ, controlled by the hyperparameter λ. This regularization limits weight complexity and mitigates overfitting to high-frequency variations in the residual component, thereby improving predictor generalization.

Algorithm 2 summarizes the training procedure based on the cached trend and residual components. The model maps the decomposition-guided trend–residual representations to future traffic flows $\hat{Y}$, linking MEMD-based representation construction with the LSTM–Bi-GRU predictor.
**Algorithm 2** Training Procedure of the Proposed Hybrid Framework**Input:** 
Training dataset $D={\left\{\left(X_{i},Y_{i}\right)\right\}}_{i=1}^{m}$, where $X_{i}\in R^{T\times N}$ and $Y_{i}\in R^{H\times N}$**Output:** 
Optimized model parameters Θ  1:Initialize parameters Θ  2:**while** not converged **do**  3:   Sample mini-batch $B={\left\{\left(X_{i},Y_{i}\right)\right\}}_{i=1}^{N}$ from D  4:   **for** each sample $(X_{i},Y_{i})\in B$ **do**  5:     $\left(C_{\mathrm{per}},C_{\mathrm{tr}}\right)\leftarrow \mathrm{RetrieveCachedTrendResidual}\left(X_{i}\right)$  6:     $H_{\mathrm{tr}}\leftarrow \mathrm{LSTM}\left(C_{\mathrm{tr}}\right)$  7:     $H_{\mathrm{per}}\leftarrow \mathrm{Bi}-\mathrm{GRU}\left(C_{\mathrm{per}}\right)$  8:     $H_{\mathrm{joint}}\leftarrow \left[H_{\mathrm{tr}}⊕H_{\mathrm{per}}\right]$  9:     $z_{i}\leftarrow \mathrm{ReLU}\left(W_{c}H_{\mathrm{joint}}+b_{c}\right)$10:     ${\hat{Y}}_{i}\leftarrow W_{f}z_{i}+b_{f}$11:   **end for**12:   $J\left(\Theta \right)\leftarrow \frac{1}{N}\sum_{i=1}^{N}∥Y_{i}-{\hat{Y}}_{i}{∥}_{2}^{2}+\lambda {∥\Theta ∥}_{2}^{2}$13:   $\Theta \leftarrow \Theta -\eta \nabla_{\Theta }J\left(\Theta \right)$14:**end while**15:**return** Θ

## 4. Experiments

### 4.1. Experimental Setup

Experiments are conducted on PeMS04 [42] and PeMS08 [43], two standard benchmarks derived from the California Department of Transportation Performance Measurement System (Caltrans PeMS). Both datasets contain multivariate traffic observations from urban sensor networks at 5 min intervals. PeMS04 includes 307 sensors and 16,992 time steps, whereas PeMS08 includes 170 sensors and 17,856 time steps.

All methods follow the same experimental protocol. Missing values are imputed before training, variables are normalized to [0,1], and all metrics are computed after transforming predictions and ground-truth values back to the original traffic-flow scale. The data are converted into supervised samples using a sliding-window scheme, with both the input length and prediction horizon set to 12 steps, corresponding to 60 min ahead forecasting. For fair comparison, all methods use the same train/validation/test split, preprocessing strategy, input length, prediction horizon, and evaluation metrics. For graph-based baselines, the node set is consistent with PeMS04 and PeMS08, and the graph structure follows the corresponding benchmark setting. The proposed model does not use an explicit adjacency matrix, road-network distance matrix, or graph-based message-passing module. The comparison therefore evaluates whether decomposition-guided temporal representation learning can achieve competitive forecasting performance under the same benchmark protocol.

For implementation efficiency, MEMD-based reconstruction is performed after sliding-window sample generation and cached before neural network training; MEMD is therefore not repeated in each training epoch. Each MEMD unit corresponds to one multichannel sliding input window, in which all sensor-flow channels are jointly processed; the channel dimensions are 307 for PeMS04 and 170 for PeMS08. The framework does not use additional periodic windows or manually defined branch-specific window ratios; both branches are driven by components reconstructed from the same 12-step input window [44].

For temporal covariate analysis, calendar-based variables are constructed from timestamps, including time-of-day and day-of-week, and encoded using sine and cosine transformations. Since the standard PeMS04 and PeMS08 benchmark files do not provide synchronized weather or event annotations, such variables are not included in the main experiments and are left for future work. Accordingly, the interpretation of the reconstructed components is based on observable flow-derived regimes, including stable, peak, and rapid-transition intervals.

Performance is evaluated by mean absolute error (MAE), root mean square error (RMSE), and mean absolute percentage error (MAPE), defined as:

(18) $$\mathrm{MAE}=\frac{1}{N}\sum_{i=1}^{N}\left|y_{i}-{\hat{y}}_{i}\right|,$$

(19) $$\mathrm{RMSE}=\sqrt{\frac{1}{N}\sum_{i=1}^{N}{\left(y_{i}-{\hat{y}}_{i}\right)}^{2}},$$

(20) $$\mathrm{MAPE}=\frac{100\%}{N}\sum_{i=1}^{N}\left|\frac{y_{i}-{\hat{y}}_{i}}{y_{i}}\right|,$$

where $y_{i}$ and ${\hat{y}}_{i}$ denote the ground-truth and predictions, respectively, and *N* is the total number of predicted points. MAPE excludes zero targets to avoid division by zero.

### 4.2. Experimental Results

On PeMS04, compared with representative recent baselines, the proposed method reduces MAE, RMSE, and MAPE by 2.04%, 1.56%, and 5.89%, respectively. On PeMS08, the corresponding relative improvements are 4.90%, 5.35%, and 3.05%. Although the absolute margins over the closest competing results are moderate, the proposed framework achieves competitive forecasting performance across the two datasets and three evaluation metrics.

Comparative analyses suggest the performance gain is not simply due to a more sophisticated predictor. Although several graph-based baselines explicitly model spatial dependencies, the proposed method focuses on decomposition-guided temporal representation learning. The improvement is more plausibly attributed to the decomposition stage, which constructs a discriminative forecasting-oriented representation by separating slowly varying trends from fast local fluctuations before recurrent modeling. This separation reduces interference between heterogeneous temporal patterns and facilitates subsequent feature learning.

### 4.3. Qualitative Forecasting Behavior

**Figure 3:**
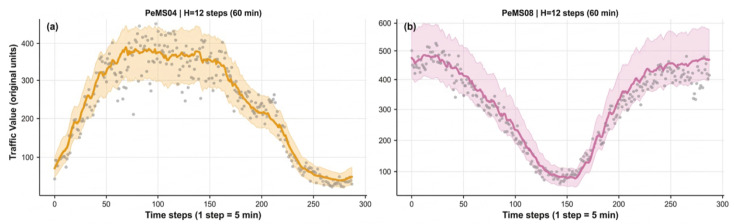
Pointforecast visualization over the first 24 h of the test set. The figure on the left (**a**) shows the predictions for peak modes on the PeMS04 test set. The figure on the right (**b**) shows the predictions for drop-off modes on the PeMS08 test set.

Beyond aggregate error statistics, it is important to examine the proposed framework can preserve temporal patterns over continuous forecasting horizons. Although Table 1 reports overall MAE, RMSE, and MAPE, these metrics do not fully show how well the model follows turning points, gradual trend variations, and rapid local fluctuations. Therefore, Figure 3 provides a qualitative point-forecast visualization over the first 24 h of the test set on PeMS04 and PeMS08, complementing the quantitative comparison.

As shown in Figure 3, the predicted series closely follows the observed series on both datasets and captures the main turning points of the daily traffic pattern. The framework is trained with an MSE-based objective and produces deterministic point forecasts, the figure should be interpreted as a point-forecast visualization. Despite differences in dataset scale and temporal profile, the two cases show consistent qualitative behavior, providing visual evidence that the proposed framework can maintain stable multistep forecasts under different traffic profiles.

To avoid relying only on qualitative visual inspection, segment-level quantitative errors during peak and rapid-transition intervals are further reported in Section 5.5.

## 5. Ablation Study

To examine whether the proposed framework functions as an integrated representation–modeling pipeline, we conduct controlled ablation studies on decomposition, IMF reconstruction, branch assignment, temporal covariates, and local transition behavior. Within each study, all variants are evaluated using the same data split, input length, prediction horizon, optimizer, and evaluation metrics. For diagnostic experiments, we focus on relative differences among variants under the same controlled protocol, rather than on direct comparisons with the main results in Table 1.

### 5.1. Ablation Study on Decomposition

This ablation examines whether MEMD-based reconstruction provides a more effective representation for multistep forecasting. Following Section 3, each traffic sequence is reorganized into a low-frequency trend component and a high-frequency fluctuation component. The trend component preserves slowly varying traffic levels, while the fluctuation component captures short-term oscillations and local deviations.

Table 2 shows that the reconstructed components are beneficial when retained together and fused appropriately. Decomp Concat and Decomp Gated both improve over the raw-input baseline on the reported metrics, whereas Decomp Attention does not yield consistent gains. Although Decomp Gated obtains the lowest MAE on PeMS08, Decomp Concat achieves the lowest RMSE on both datasets and the lowest MAE on PeMS04 with a simpler fusion head. This suggests that the main benefit comes from representation reorganization rather than a more expressive fusion mechanism.

**Figure 4:**
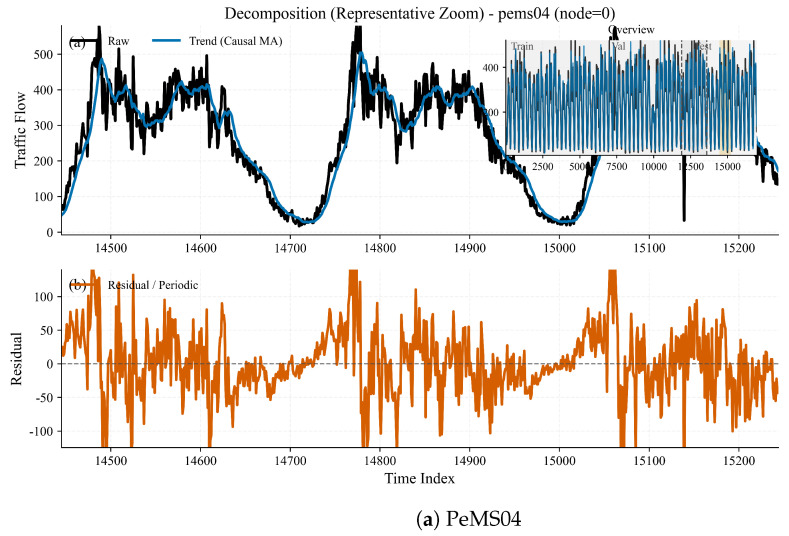

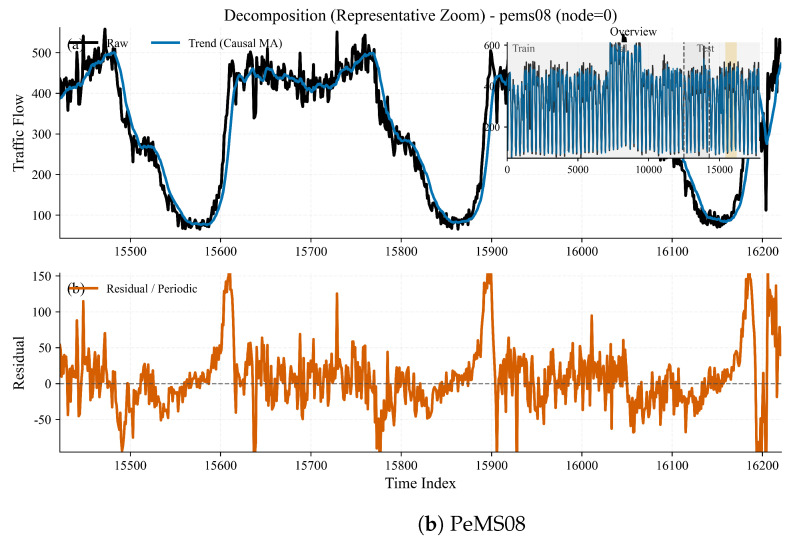
Representativedecomposition examples at node 0 on PeMS04 and PeMS08.

As shown in Figure 4, the trend component follows the dominant level evolution of the original signal, whereas the fluctuation component concentrates faster local variations. From a traffic perspective, the trend component reflects the slowly varying demand or congestion baseline, while the fluctuation component becomes more pronounced around short-term departures from this baseline. Modeling either component alone is insufficient: Trend Only tends to oversmooth short-term changes, while Fluctuation Only lacks the traffic-level information required for stable extrapolation.

### 5.2. Ablation Study on IMF Reconstruction

This reconstruction ablation uses a controlled diagnostic setting that differs from the full benchmark evaluation in Section 4 in model configuration and training setup, while keeping the data split, input length, prediction horizon, and evaluation metrics fixed across all reconstruction variants. Its purpose is to compare IMF grouping strategies under the same ablation protocol, rather than to provide a directly comparable estimate of the final benchmark performance reported in Table 1.

As shown in Table 3, IMF-wise modeling improves over the raw-input setting, confirming that decomposed modes contain useful predictive information. However, modeling each IMF separately also introduces redundancy. The proposed two-group reconstruction achieves the lowest MAE and RMSE, while the three-group setting obtains a slightly lower MAPE. Considering absolute accuracy and sensitivity to large deviations, the two-component design provides the most balanced overall profile.

### 5.3. Ablation Study on Branch Assignment

This ablation evaluates whether the two reconstructed components benefit from different temporal encoders. This experiment follows the main evaluation protocol used in Section 4. All variants use the same MEMD-based reconstruction and training protocol; only the encoder assignment is changed.

Table 4 shows that the LSTM–Bi-GRU assignment obtains the lowest errors across all three metrics. This matches the temporal roles of the reconstructed components: the trend component benefits from the LSTM cell state, which preserves slowly varying levels, while the fluctuation component benefits from Bi-GRU encoding of local temporal context within the observed window. The backward direction does not access future target values; it only encodes the historical input in reverse order. The weaker performance of homogeneous designs further supports component-specific encoder assignment.

### 5.4. Ablation Study on Temporal Variables

This ablation evaluates calendar-based covariates derived from timestamps, including time-of-day, day-of-week, and weekend indicators. It should be noted that external factors such as weather conditions, traffic incidents, and special events may also affect traffic flow evolution. Unfortunately, the standard PeMS04 and PeMS08 benchmark files do not provide synchronized sensor-level annotations for these variables. Therefore, the present study focuses on timestamp-derived temporal covariates, while incorporating richer external context is left for future work.

Table 5 shows that temporal covariates provide auxiliary gains, especially when intra-day information is included. Time-of-day clearly improves over the flow-only setting, while day-of-week alone improves MAPE but not MAE or RMSE. The combination of time-of-day, day-of-week, and weekend indicators yields the lowest MAE and RMSE. These results suggest that temporal covariates are helpful but secondary to the decomposition-guided representation.

### 5.5. Ablation Study on Local Transition Behavior

Aggregate metrics do not fully reveal model behavior around turning points, peak states, and rapid transitions. We, therefore, examine local forecasting behavior using zoomed-in predictions and a regime-wise diagnostic analysis.

**Figure 5:**
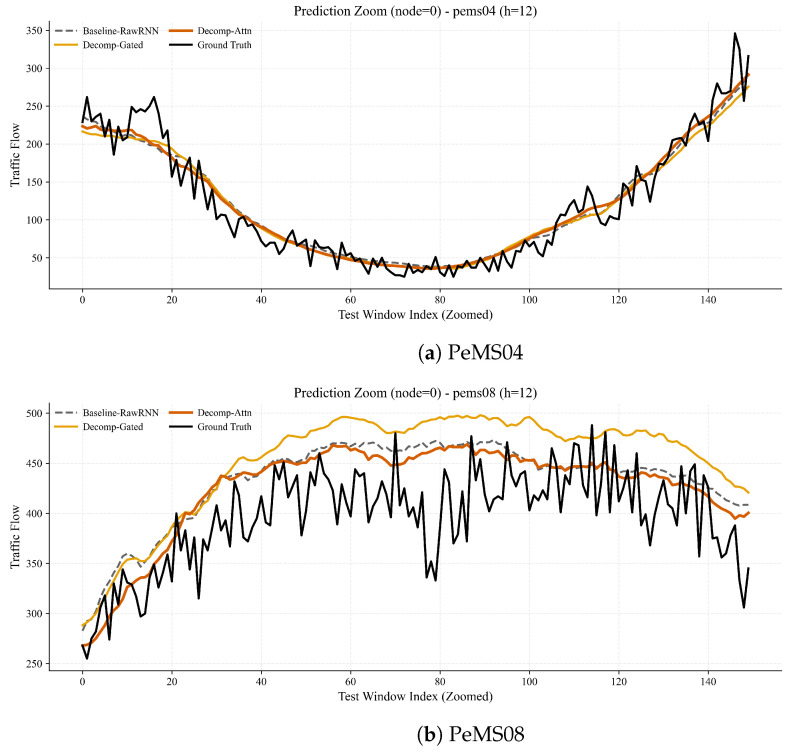
Prediction zoom at node 0 on PeMS04 and PeMS08.

Figure 5 shows that the proposed model follows local reversals more closely than the raw-input baseline, especially near turning points and recovery phases. This behavior is consistent with the roles of the reconstructed components: the trend branch anchors the forecast to the underlying traffic level, while the fluctuation branch provides short-term corrective information when the observed state departs from its local baseline.

Table 6 presents a representative diagnostic analysis on PeMS04 under three flow-derived regimes. Stable intervals are defined by the lowest quartile of absolute first-order temporal differences, peak intervals by the highest quartile of flow values, and rapid-transition intervals by the highest quartile of absolute first-order temporal differences. When an interval satisfies multiple criteria, labels are assigned in the order of rapid transition, peak, and stable. The relative gain is computed as (Baseline−Ours)/Baseline×100%. The proposed model reduces errors in all regimes, with the largest gains under rapid transitions. This pattern supports the trend–residual interpretation: the trend representation provides a stable reference for regular and high-demand states, whereas the residual representation is more effective when traffic states change abruptly around the local baseline. These results show that decomposition-guided representation improves not only overall accuracy but also local forecasting behavior during abrupt traffic changes.

Overall, the ablation studies indicate that the gains mainly come from decomposition-guided representation learning and component-specific temporal modeling. MEMD-based reconstruction yields a compact trend–residual representation, and the heterogeneous LSTM–Bi-GRU assignment better matches the temporal properties of the reconstructed components. Taken together, these results suggest that the gains are mainly attributable to the trend–residual representation and the component-specific branch assignment.

## 6. Conclusions and Future Work

This paper presents a decomposition-guided dual-branch recurrent framework for multistep traffic flow forecasting. MEMD is used to construct aligned trend and residual representations from multiscale traffic components, which are then modeled by LSTM and Bi-GRU branches, respectively. Experiments on PeMS04 and PeMS08 show competitive forecasting performance, and the ablation studies further indicate that the gains mainly come from trend–residual representation learning and component-specific temporal modeling rather than increased fusion complexity. These results suggest that organizing heterogeneous traffic observations into structured trend and residual representations can improve multistep forecasting.

Future work will incorporate explicit spatial dependency modeling, improve the efficiency of MEMD-based reconstruction for online inference, and evaluate the framework under more diverse traffic scenarios.

## Figures and Tables

**Table 1:** Comparison with diverse forecasting baselines on PeMS04 and PeMS08. ↓ indicates that a smaller value means better performance.

Method	PeMS04			PeMS08		
	MAE ↓	RMSE ↓	MAPE (%) ↓	MAE ↓	RMSE ↓	MAPE (%) ↓
HA [45]	24.50	39.83	16.60	21.19	36.64	13.82
VAR [46]	21.87	32.26	15.70	18.66	27.35	12.81
LSTM [47]	21.37	33.31	15.21	17.38	26.27	12.63
DCRNN [15]	24.71	38.12	17.12	17.86	28.83	12.45
STGCN [14]	22.70	35.55	14.59	18.61	28.16	13.12
Graph WaveNet [16]	20.65	33.08	14.66	16.23	25.02	12.43
MTGNN [48]	20.08	32.56	13.96	16.39	25.93	10.17
STFGNN [49]	20.83	32.09	14.02	16.46	25.81	10.92
STGNCDE [50]	20.21	32.09	13.76	16.45	25.81	10.92
DMSTGCN [51]	23.59	36.83	16.43	18.65	29.14	12.01
D2STGNN [52]	20.55	32.99	13.82	16.69	26.41	11.17
ASTGCN [20]	22.93	35.22	16.56	18.61	28.16	13.08
GMAN [21]	20.14	32.60	14.20	16.31	25.92	11.13
STID [53]	20.58	32.79	14.38	16.58	26.89	11.33
Traffic Transformer [22]	19.75	32.35	12.82	15.79	24.88	9.93
**Proposed (Ours)**	**19.67**	**31.59**	**12.95**	**15.51**	**24.43**	**9.86**

**Table 2:** Ablation study on decomposition on PeMS04 and PeMS08. ↓ indicates that a smaller value means better performance.

Model	PeMS04		PeMS08	
	RMSE ↓	MAE ↓	RMSE ↓	MAE ↓
Baseline	38.71	23.79	33.25	21.40
Trend Only	39.11	24.21	33.95	22.01
Fluctuation Only	49.30	30.05	44.46	30.08
Decomp Concat	**38.35**	**23.34**	**33.03**	21.03
Decomp Gated	38.57	23.56	33.12	**20.92**
Decomp Attention	39.26	24.30	33.25	21.29

**Table 3:** Ablation study on IMF reconstruction on PeMS04. ↓ indicates that a smaller value means better performance.

Reconstruction Strategy	MAE ↓	RMSE ↓	MAPE (%) ↓
Raw Input	26.23±0.06	40.15±0.01	19.59±0.93
IMF-wise Modeling	23.34±0.35	33.72±0.53	18.83±0.14
**Two Groups (Ours)**	18.05±0.11	28.42±0.19	14.23±0.16
Three Groups	18.89±0.21	29.65±0.28	14.09±0.14
Four Groups	20.01±0.04	33.17±0.05	14.84±0.37

**Table 4:** Ablation study on branch assignment on PeMS04. ↓ indicates that a smaller value means better performance.

Architecture	MAE ↓	RMSE ↓	MAPE (%) ↓
LSTM–LSTM	21.02±0.16	33.89±0.24	14.36±1.03
GRU–GRU	20.88±0.12	34.05±0.13	13.99±0.11
Bi-GRU–Bi-GRU	20.69±0.25	33.66±0.30	13.54±0.11
LSTM–GRU	20.45±0.30	33.87±0.32	13.56±0.58
GRU–Bi-GRU	20.23±0.07	32.69±0.42	13.69±0.22
Transformer–Transformer	20.77±0.25	33.80±0.21	14.02±0.16
**LSTM–Bi-GRU (Ours)**	19.67±0.11	31.59±0.08	12.95±0.03

**Table 5:** Ablation study on temporal variables on PeMS04. ↓ indicates that a smaller value means better performance.

Input Setting	MAE ↓	RMSE ↓	MAPE (%) ↓
Flow Only	28.06±0.45	42.96±0.76	26.16±0.66
Flow + Time-of-day	26.15±0.12	40.15±0.31	20.04±0.71
Flow + Day-of-week	28.25±0.37	43.04±0.44	23.49±1.75
Flow + Time + Day-of-week	26.15±0.58	40.38±0.72	19.51±1.02
**Flow + Time + Day-of-week + Weekend**	25.33±0.17	39.15±0.22	20.21±1.20

**Table 6:** Segment-wise error comparison under different traffic regimes on PeMS04. ↓ indicates that a smaller value means better performance.

Regime	Model	MAE ↓	RMSE ↓	MAPE (%) ↓
Stable	Baseline	15.39	26.28	22.61
	Ours	**12.47**	**21.10**	**19.41**
	Gain (%)	18.96	19.71	14.15
Peak	Baseline	41.96	57.77	10.30
	Ours	**34.04**	**47.88**	**8.42**
	Gain (%)	18.88	17.13	18.30
Rapid Transition	Baseline	41.24	56.34	17.32
	Ours	**31.79**	**45.07**	**12.91**
	Gain (%)	22.91	20.01	25.44

## Data Availability

All original research contributions presented in this study have been included in the paper. For further inquiries, please contact the corresponding author.

## References

[1] Zhou Y. (2025). An urban traffic flow prediction method based on multi-source data fusion. Proceedings of the 2025 International Conference on Software Engineering and Computer Applications.

[2] Diao C., Zhang D., Liang W., Jiang M., Li K. (2024). A novel attention-based dynamic multi-graph spatial-temporal graph neural network model for traffic prediction. IEEE Trans. Emerg. Top. Comput. Intell..

[3] Chen Y., Shu T., Zhou X., Zheng X., Kawai A., Fueda K., Yan Z., Liang W., Wang K.I.K. (2022). Graph attention network with spatial-temporal clustering for traffic flow forecasting in intelligent transportation system. IEEE Trans. Intell. Transp. Syst..

[4] Ye B.L., Zhang M., Li L., Liu C., Wu W. (2024). A survey of traffic flow prediction methods based on long short-term memory networks. IEEE Intell. Transp. Syst. Mag..

[5] Gao D., Li P., Wang M., Liang Y., Liu S., Zhou J., Wang L., Zhang Y. (2023). CSF-GTNet: A novel multi-dimensional feature fusion network based on Convnext-GeLU-BiLSTM for EEG-signals-enabled fatigue driving detection. IEEE J. Biomed. Health Inform..

[6] Qian W., Zhao Y., Zhang D., Chen B., Zheng K., Zhou X. (2023). Towards a unified understanding of uncertainty quantification in traffic flow forecasting. IEEE Trans. Knowl. Data Eng..

[7] Zeng A., Chen M., Zhang L., Xu Q. (2023). Are transformers effective for time series forecasting?. Proceedings of the AAAI Conference on Artificial Intelligence.

[8] Hu H.X., Hu Q., Tan G., Zhang Y., Lin Z.Z. (2023). A multi-layer model based on transformer and deep learning for traffic flow prediction. IEEE Trans. Intell. Transp. Syst..

[9] Zhang J., Mao S., Yang L., Ma W., Li S., Gao Z. (2024). Physics-informed deep learning for traffic state estimation based on the traffic flow model and computational graph method. Inf. Fusion.

[10] Huang Y., Hasan N., Deng C., Bao Y. (2022). Multivariate empirical mode decomposition based hybrid model for day-ahead peak load forecasting. Energy.

[11] Chen Z., Lu Z., Chen Q., Zhong H., Zhang Y., Xue J., Wu C. (2022). Spatial–temporal short-term traffic flow prediction model based on dynamical-learning graph convolution mechanism. Inf. Sci..

[12] Al-Selwi H.F., Abd Aziz A.B., Abas F.S., Hamzah N.A.A., Mahmud A.B. (2022). The impact of weather data on traffic flow prediction models. IAES Int. J. Artif. Intell..

[13] Fan J., Zhu F., Weng W., Zhang X., Jiang H., Tian H., Wu H. (2025). Dynamic modeling and analysis of Bi-directional traffic flows through a deep spatio-temporal graph neural network. IEEE Trans. Big Data.

[14] Wang C., Zhang K., Wang H., Chen B. (2023). Auto-STGCN: Autonomous spatial-temporal graph convolutional network search. ACM Trans. Knowl. Discov. Data.

[15] Huang Y., Weng Y., Yu S., Chen X. (2019). Diffusion convolutional recurrent neural network with rank influence learning for traffic forecasting. Proceedings of the 2019 18th IEEE International Conference on Trust, Security and Privacy in Computing and Communications/13th IEEE International Conference on Big Data Science and Engineering (TrustCom/BigDataSE).

[16] Wu Z., Pan S., Long G., Jiang J., Zhang C. (2019). Graph WaveNet for Deep Spatial-Temporal Graph Modeling. Proceedings of the 28th International Joint Conference on Artificial Intelligence (IJCAI-19); International Joint Conference on Artificial Intelligence (IJCAI).

[17] Zhao L., Song Y., Zhang C., Liu Y., Wang P., Lin T., Deng M., Li H. (2019). T-GCN: A temporal graph convolutional network for traffic prediction. IEEE Trans. Intell. Transp. Syst..

[18] Song C., Lin Y., Guo S., Wan H. (2020). Spatial-Temporal Synchronous Graph Convolutional Networks: A New Framework for Spatial-Temporal Network Data Forecasting. Proceedings of the AAAI Conference on Artificial Intelligence.

[19] Bai L., Yao L., Li C., Wang X., Wang C. (2020). Adaptive graph convolutional recurrent network for traffic forecasting. Adv. Neural Inf. Process. Syst..

[20] Guo S., Lin Y., Feng N., Song C., Wan H. (2019). Attention Based Spatial-Temporal Graph Convolutional Networks for Traffic Flow Forecasting. Proceedings of the AAAI Conference on Artificial Intelligence.

[21] Zheng C., Fan X., Wang C., Qi J. (2020). GMAN: A Graph Multi-Attention Network for Traffic Prediction. Proceedings of the AAAI Conference on Artificial Intelligence.

[22] Cai L., Janowicz K., Mai G., Yan B., Zhu R. (2020). Traffic transformer: Capturing the continuity and periodicity of time series for traffic forecasting. Trans. GIS.

[23] Wu H., Xu J., Wang J., Long M. (2021). Autoformer: Decomposition transformers with auto-correlation for long-term series forecasting. Adv. Neural Inf. Process. Syst..

[24] Jiang J., Han C., Zhao W.X., Wang J. (2023). PDFormer: Propagation Delay-Aware Dynamic Long-Range Transformer for Traffic Flow Prediction. Proceedings of the AAAI Conference on Artificial Intelligence.

[25] Liu H., Dong Z., Jiang R., Deng J., Deng J., Chen Q., Song X. (2023). Spatio-temporal adaptive embedding makes vanilla transformer sota for traffic forecasting. Proceedings of the 32nd ACM International Conference on Information and Knowledge Management.

[26] Pan Z., Liang Y., Wang W., Yu Y., Zheng Y., Zhang J. (2019). Urban traffic prediction from spatio-temporal data using deep meta learning. Proceedings of the 25th ACM SIGKDD International Conference on Knowledge Discovery & Data Mining.

[27] Wang Y., Zhang D., Liu Y., Dai B., Lee L.H. (2019). Enhancing transportation systems via deep learning: A survey. Transp. Res. Part C Emerg. Technol..

[28] Tedjopurnomo D.A., Bao Z., Zheng B., Choudhury F.M., Qin A.K. (2020). A survey on modern deep neural network for traffic prediction: Trends, methods and challenges. IEEE Trans. Knowl. Data Eng..

[29] Fafoutellis P., Vlahogianni E.I. (2025). A theory-informed multivariate causal framework for trustworthy short-term urban traffic forecasting. Transp. Res. Part C Emerg. Technol..

[30] Wang R., Xin Y., Zhang Y., Perez-Cruz F., Raubal M. (2025). Counterfactual explanations for deep learning-based traffic forecasting. Commun. Transp. Res..

[31] Kong L., Yang H., Li W., Zhang Y., Guan J., Zhou S. (2024). Traffexplainer: A framework toward gnn-based interpretable traffic prediction. IEEE Trans. Artif. Intell..

[32] Chen J., Zheng L., Hu Y., Wang W., Zhang H., Hu X. (2024). Traffic flow matrix-based graph neural network with attention mechanism for traffic flow prediction. Inf. Fusion.

[33] Yang H.F., Chen Y.P.P. (2019). Hybrid deep learning and empirical mode decomposition model for time series applications. Expert Syst. Appl..

[34] Tan Z., Shi Y., Zhang Y. (2025). Traffic Flow Prediction Based on Multimodal Spatio-Temporal Bayesian Neural Network. Proceedings of the International Conference on Information, Computing and Technology.

[35] Sun R., Cheng N., Li C., Quan W., Zhou H., Wang Y., Zhang W., Shen X. (2025). A comprehensive survey of knowledge-driven deep learning for intelligent wireless network optimization in 6G. IEEE Commun. Surv. Tutor..

[36] Wang L., He H., Dong Y., Li X., Gan W., Zhang X. (2026). Predicting street-level distribution of bike-sharing traffic volume in metro station areas using integrated generative adversarial networks. J. Transp. Geogr..

[37] Ma C., Zhao Y., Dai G., Xu X., Wong S.C. (2022). A novel STFSA-CNN-GRU hybrid model for short-term traffic speed prediction. IEEE Trans. Intell. Transp. Syst..

[38] Deng C., Huang Y., Hasan N., Bao Y. (2022). Multi-step-ahead stock price index forecasting using long short-term memory model with multivariate empirical mode decomposition. Inf. Sci..

[39] Ur Rehman N., Mandic D.P. (2011). Filter bank property of multivariate empirical mode decomposition. IEEE Trans. Signal Process..

[40] Naheliya B., Redhu P., Kumar K. (2024). Bi-directional long short term memory neural network for short-term traffic speed prediction using gravitational search algorithm. Int. J. Intell. Transp. Syst. Res..

[41] Ma X., Tao Z., Wang Y., Yu H., Wang Y. (2015). Long short-term memory neural network for traffic speed prediction using remote microwave sensor data. Transp. Res. Part C Emerg. Technol..

[42] Xiao J., Huang Y. (2025). Traffic state identification method based on GA-EWFCM. Proceedings of the Tenth International Conference on Electromechanical Control Technology and Transportation (ICECTT 2025).

[43] Wu L., Li S., Li H., Huang J., Lei X., Jiang H. (2025). Spatio-temporal Transfer Learning for Urban Data Modeling. Proceedings of the 2025 IEEE 28th International Conference on Computational Science and Engineering (CSE).

[44] Lin S., Lin W., Wu W., Zhao F., Mo R., Zhang H. (2025). Segrnn: Segment recurrent neural network for long-term time series forecasting. IEEE Internet Things J..

[45] Lei Z., Dong Y., Li J., Chen C. (2025). St-fit: Inductive spatial-temporal forecasting with limited training data. Proceedings of the AAAI Conference on Artificial Intelligence.

[46] Sims C.A. (1980). Macroeconomics and reality. Econometrica: Journal of the Econometric Society.

[47] Greff K., Srivastava R.K., Koutník J., Steunebrink B.R., Schmidhuber J. (2016). LSTM: A search space odyssey. IEEE Trans. Neural Netw. Learn. Syst..

[48] Gao J., Zhang X., Tian L., Liu Y., Wang J., Li Z., Hu X. (2022). MTGNN: Multi-task graph neural network based few-shot learning for disease similarity measurement. Methods.

[49] Li M., Zhu Z. (2021). Spatial-Temporal Fusion Graph Neural Networks for Traffic Flow Forecasting. Proceedings of the AAAI Conference on Artificial Intelligence.

[50] Choi J., Choi H., Hwang J., Park N. (2022). Graph Neural Controlled Differential Equations for Traffic Forecasting. Proceedings of the AAAI Conference on Artificial Intelligence.

[51] Han L., Du B., Sun L., Fu Y., Lv Y., Xiong H. (2021). Dynamic and multi-faceted spatio-temporal deep learning for traffic speed forecasting. Proceedings of the 27th ACM SIGKDD Conference on Knowledge Discovery & Data Mining.

[52] Shao Z., Zhang Z., Wei W., Wang F., Xu Y., Cao X., Jensen C.S. (2022). Decoupled dynamic spatial-temporal graph neural network for traffic forecasting. Proc. VLDB Endow..

[53] Shao Z., Zhang Z., Wang F., Wei W., Xu Y. (2022). Spatial-temporal identity: A simple yet effective baseline for multivariate time series forecasting. Proceedings of the 31st ACM International Conference on Information & Knowledge Management.

